# Association between epidural analgesia and indications for intrapartum caesarean delivery in group 1 of the 10-group classification system at a tertiary maternity hospital, Shanghai, China: a retrospective cohort study

**DOI:** 10.1186/s12884-021-03925-z

**Published:** 2021-06-29

**Authors:** Rong Lin, Peng Shi, Haibing Li, Zhiqiang Liu, Zhendong Xu

**Affiliations:** 1 Department of Anesthesiology, Shanghai First Maternity and Infant Hospital, Tongji University School of Medicine, No. 2699 W Gaoke Rd, Shanghai, 201024 China; 2 Department of Statistics and Data Management, Children’s Hospital of Fudan University, No. 399 Wanyuan Rd, Shanghai, 201102 China; 3 Center of Evidence-based Medicine, Fudan University, No. 180 Fenglin Rd, Shanghai, 200032 China

**Keywords:** Caesarean delivery, Epidural anaesthesia, Intrapartum, Nulliparous women, Labour pain

## Abstract

**Background:**

In this study, we aimed to determine whether epidural analgesia affects the indications for intrapartum caesarean delivery, such as foetal distress, dystocia, or maternal request, in nulliparous term women with spontaneous labour (Group 1 in the 10-Group Classification System).

**Methods:**

We conducted a retrospective cohort study and collected data from the electronic medical records of deliveries performed in our institution between 1 January 2017 and 30 June 2017. Women conforming to the criterion of Group 1 according to the 10-Group Classification System were enrolled. We compared labour outcomes between women with and without epidural analgesia and analysed the association between epidural analgesia and indications for caesarean by using multivariate logistic regression analysis.

**Results:**

A total of 3212 women met the inclusion criteria, and 2876 were enrolled in the final analyses. Women who received epidural analgesia had a significantly lower intrapartum caesarean delivery rate (16.0% vs. 26.7%, *P* < 0.001), higher rates of amniotomy (53.4% vs. 42.3%, *P* < 0.001) and oxytocin augmentation (79.5% vs. 67.0%, *P* < 0.001), and a higher incidence of intrapartum fever (≥38 °C) (23.3% vs. 8.5%, *P* < 0.001) than those who did not receive epidural analgesia. There were no significant differences between the groups for most indications, except a lower probability of maternal request for caesarean delivery (3.9% vs. 10.5%, *P* < 0.001) observed in women who received epidural analgesia than in those who did not. Epidural analgesia was revealed to be associated with a decreased risk of maternal request for caesarean delivery (adjusted odds ratio [aOR], 0.30; 95% confidence interval [CI], 0.22–0.42; *P* < 0.001); however, oxytocin augmentation was related to an increased risk of maternal request (aOR, 2.34; 95%CI, 1.47–3.75; *P* < 0.001). Regarding the reasons for the maternal request for caesarean delivery, significantly fewer women complained of pain (0.5% vs. 4.6%, *P* < 0.001) or had no labour progress (1.3% vs. 3.6%, *P* < 0.001) among those who received analgesia.

**Conclusions:**

Among the women in Group 1, epidural analgesia was associated with a lower intrapartum caesarean delivery rate, which may be explained by a reduction in the risk of maternal request for an intrapartum caesarean delivery.

## Background

As the recognised gold standard for pain relief during labour and delivery, epidural analgesia (EA) is widely used for women in labour worldwide. A recent systematic review in the Cochrane Database of Systematic Reviews, comprising 40 trials and involving over 11,000 women, declared that EA has no impact on the risk of caesarean delivery (CD) [1]. However, the indications for intrapartum CD in women under EA have not been adequately evaluated. It is unclear whether EA is a risk factor for specific indications of CD during labour, such as foetal distress, dystocia, or maternal request for CD. In addition, it should be noted that the included trials did not analyse data according to maternal characteristics. Focusing only on the overall effects of EA, regardless of intrapartum management and epidemiological parameters, is inadvisable. It is reasonable to presume that EA administration may have a different impact on intrapartum CD in different obstetric populations.

The 10-Group Classification System (TGCS) [2], originally designed to audit and achieve justified CD rates, has been extensively recommended by the World Health Organisation [3]. The TGCS divides women in labour into 10 groups according to six obstetric characteristics or concepts, including parity, singleton or multiple pregnancy, gestational age, foetal presentation, history of CD, and labour process (Table 1), allowing an apples-to-apples comparison among different institutions and regions. The TGCS uses a rigorous and standard approach in elucidating results, and is considered useful in further analyses of all perinatal events and outcomes [4], not just in the control of CD rates [5, 6]. However, the TGCS has rarely been applied to evaluate the effects of EA on labour outcomes.

**Table 1:** The 10-Group Classification System

Group 1	Nulliparous, single cephalic, ≥37 weeks, spontaneous labour
Group 2	Nulliparous, single cephalic, ≥37 weeks, induced or CD before labour
Group 3	Multiparous (excluding previous CD), single cephalic, ≥37 weeks, spontaneous labour
Group 4	Multiparous (excluding previous CD), single cephalic, ≥37 weeks, induced or CD before labour
Group 5	Previous CD, single cephalic, ≥37 weeks
Group 6	All nulliparous breeches
Group 7	All multiparous breeches (including previous CD)
Group 8	All multiple pregnancies (including previous CD)
Group 9	All abnormal lies (including previous CD)
Group 10	All single cephalic, ≤36 weeks (including previous CD)

*Abbreviation*: *CD* caesarean delivery

The increasing global rates of CD over the decades have been an important public health concern [7]. In China, the rates of CD have grown considerably from 28.8% in 2008 to 36.7% in 2018 [8]. Moreover, repeat CD (RCD) remains the largest contributor to the overall CD rate. Between 1979 and 2010, the RCD in the US increased by 178% [9]. Furthermore, with the implementation of the two-child policy in China, a growing number of women with a history of CD are getting pregnant again [10]. A recent cross-sectional survey has shown that the overall CD rate in Shanghai in 2016 was 41.5%, and 96.6% of women with a previous CD had RCDs [11]. Compared with previous vaginal delivery, RCDs are associated with a significantly increased risk of complications, such as uterine rupture, placenta previa, placenta accreta, and the need for hysterectomy [12]. Undoubtedly, controlling the primary CD rate is of high priority in promoting maternal and foetal health. More attention should be paid to preventing primary CD, particularly in nulliparous women with spontaneous labour (Group 1 in the TGCS). An analysis of the risk factors related to indications for intrapartum CD, within specific TGCS categories, may be beneficial in preventing primary CDs in nulliparous women [13].

Therefore, we performed a retrospective cohort study to assess the association between EA and the indications for intrapartum CD in nulliparous women with spontaneous labour (Group 1 in the TGCS).

## Methods

### Study population

**Fig. 1:**
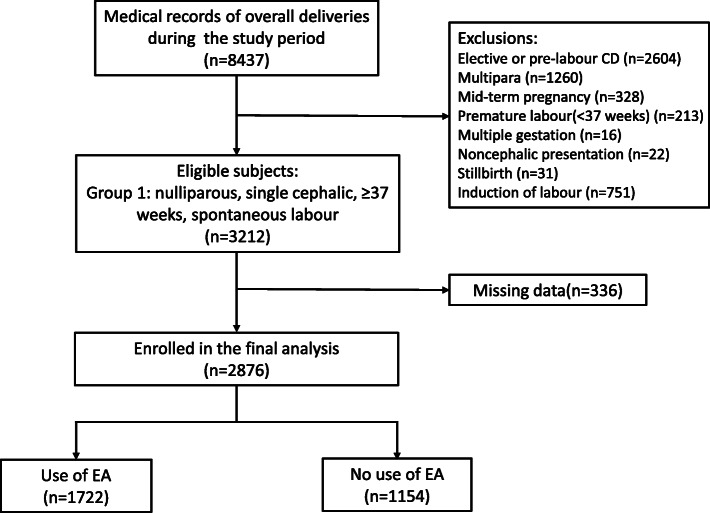
Flow diagram of the study. Abbreviations: CD, caesarean delivery; EA, epidural analgesia

The electronic medical records of all 8437 deliveries performed at Shanghai First Maternity and Infant Hospital between 1 January 2017 and 30 June 2017 were reviewed and screened for eligibility. Exclusion criteria included the following: induction of labour, pre-labour or elective CD, multiparity, preterm gestation (< 37 weeks), stillbirth, multiple gestation, and noncephalic presentation. Finally, nulliparous women meeting the criteria of Group 1 according to the TGCS were enrolled (Fig. 1).

### Prenatal management and definitions

Shanghai First Maternity and Infant Hospital is a tertiary care academic hospital, averaging 20,000 deliveries annually in recent years. All pregnancies were assessed using routine obstetrical examinations to ensure appropriate prenatal management according to institutional protocols.

When the women in labour entered the pre-delivery unit, maternal conditions (non-invasive blood pressure, electrocardiogram, pulse oxygen saturation, and temperature) were continuously monitored; additionally, the foetal status was assessed by continuous cardiotocography. Once the cervix was dilated to 2 cm, the women were sent to the delivery room and one-on-one doula service was provided. The midwife established routine monitoring and peripheral intravenous infusion and assessed pain intensity on a 100-mm visual analogue scale (VAS). If a woman requested pain relief at this time, EA was performed after the anaesthesiologist evaluated its suitability. In the absence of epidural contraindications, epidural catheterisation was established at the L3-L4 or L2-L3 interspace. After an 8- to 10-mL initial loading dose of 0.1% ropivacaine and 0.3 μg/mL sufentanil was administered, a patient-controlled EA pump was applied to maintain analgesia, with a continuous infusion of the same mixture at 8 mL/h. During analgesia, the VAS pain score was assessed every hour to ensure the quality of analgesia. If the VAS pain score was > 50 mm, a patient-controlled EA bolus dose of 5 mL was supplemented, with a 15-min lockout interval. If breakthrough pain was not ameliorated by patient-controlled EA, manual epidural provider boluses of 5 mL of 0.125% ropivacaine were provided. Maternal satisfaction with EA was also assessed with a 100-mm VAS on postpartum day 1.

Intrapartum fever was defined as body temperature ≥ 38.0 °C during labour. Decisions regarding on how to diagnose labour, how and when to accelerate the course of labour with artificial rupture of membranes or oxytocin administration, and when to carry out an intrapartum CD were made by the obstetricians, based on the local labour management guidelines. Vaginal examinations were performed every 2 h to assess the progress in labour. Amniotomy was performed if the labour failed to progress due to a lack of spontaneous rupture of the membranes. Oxytocin was administered to accelerate uterine activity with a dose of 5 mU/min increasing to a maximum dose of 30 mU/min. The oxytocin was adjusted by 5 mU/min, according to uterine activity, at 15-min intervals.

**Fig. 2:**
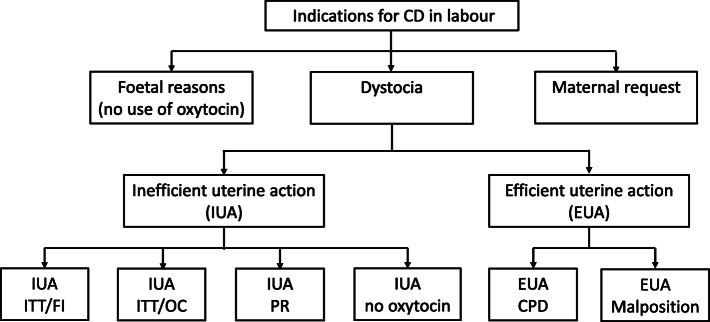
Classification of indications for intrapartum caesarean delivery. Abbreviations: CD, caesarean delivery; IUA, inefficient uterine action; EUA, efficient uterine action; ITT, inability to treat adequately with oxytocin; FI, foetal intolerance; OC, uterus over-contracting; PR, poor response to oxytocin; CPD, cephalopelvic disproportion

All indications for CD were recorded by the obstetricians according to the standard defined classification for intrapartum CD [14] (Fig. 2). The indications for CD performed during spontaneous labour were divided into foetal reasons, dystocia, and maternal request for CD. Foetal reasons were defined as suspected foetal distress without oxytocin use. Dystocia signified that the labour failed to progress and was subdivided into dystocia/inefficient uterine action (IUA) [labour progress at < 1 cm/h] and dystocia/efficient uterine action (EUA) [labour progress at > 1 cm/h]. Dystocia/inefficient uterine action was further subdivided into 1) inability to treat adequately with oxytocin due to foetal intolerance (Dys/IUA/ITT/FI); 2) inability to treat adequately with oxytocin due to uterus over-contracting (Dys/IUA/ITT/OC); 3) poor response to oxytocin (Dys/IUA/PR); and 4) no use of oxytocin (Dys/IUA/no oxytocin). Additionally, EUA was further subdivided into cephalopelvic disproportion (Dys/EUA/CPD) or malposition (Dys/EUA/malposition). If there was no other medical reason for a CD, the indication was recorded as ‘maternal request’; the reasons for the maternal request were determined by inquiry and recorded.

### Data collection

The following data were collected from the records of the women enrolled into the final analysis: maternal age, gestational age, height, weight, body mass index (BMI), abdominal circumference (AC), administration of EA (yes/no), delivery mode (intrapartum CD/assisted vaginal delivery), duration of labour (first and second stages), premature rupture of membranes (PROM) (yes/no), acceleration of labour course by amniotomy or oxytocin administration, intrapartum fever (yes/no), VAS pain score during analgesia, EA satisfaction score, indications for CD, blood loss during the procedure, birthweight, Apgar scores, and the duration of hospitalisation.

All data were extracted from the medical records by two investigators. After one investigator completed data acquisition, the other investigator cross-checked the abstracted data to guarantee its reliability and consistency. Any discrepancies were resolved by consensus.

### Statistical analyses

Continuous variables with normal distribution (as assessed by the Shapiro-Wilk method) are presented as the mean ± standard deviation and were compared using the Student’s t-test for independent samples. Due to a non-normal distribution, gestational age, VAS pain score, EA satisfaction score, blood loss during the procedure, birthweight, and the duration of hospitalisation are expressed as the median (interquartile range), and were compared using the Mann-Whitney U test. Categorical variables are presented as numbers (percentages) and were compared using the Pearson’s chi-squared or Fisher’s exact test. By comparing the rates of indications for CD in nulliparous women with and without EA, the indications for EA could be identified. Multivariate logistic regression analysis was further used to evaluate the association between EA and indications of intrapartum CD. All analyses were conducted using SPSS for Windows version 23.0 (IBM Corp., Armonk, NY). A two-sided *P* value < 0.05 was considered as statistically significant.

### Ethical approval

This retrospective, observational cohort study was approved by the Ethics Committee of Shanghai First Maternity and Infant Hospital, Shanghai, China (Ethics No.: KS1990, October 20, 2019). Due to the nature of the study, the requirement for written informed consent was waived. The study followed the Strengthening the Reporting of Observational Studies in Epidemiology statement.

## Results

Between 1 January 2017 and 30 June 2017, a total of 3212 women met the criteria for Group 1 classification in the TGCS; among them, 336 were excluded due to missing or incomplete data. A total of 2876 women were enrolled in the final analyses (Fig. 1).

Table 2 summarises the maternal demographic and pregnancy characteristics of the nulliparous women according to EA administration during labour; there were no significant differences in the characteristics between the two groups (with EA vs. without EA).

**Table 2:** Demographic and characteristics of women in the two groups

Characteristics	Use of EA (*n* = 1722)	No use of EA (*n* = 1154)	*P* value
Maternal age (y)	30.47 ± 2.97	30.67 ± 3.08	0.068
Gestational age (w)	40.0 [39.3, 40.6]	39.9 [39.1, 40.4]	0.077
Weight (kg)	70.29 ± 8.53	71.64 ± 8.74	0.060
Height (m)	1.60 ± 0.08	1.60 ± 0.04	0.118
BMI (kg/m^2^)	28.13 ± 11.89	27.82 ± 3.77	0.681
AC (cm)	101.81 ± 4.70	102.17 ± 4.78	0.364

Data were presented as mean ± standard deviation, n (%) or median [interquartile range]

*Abbreviations*: *EA* epidural analgesia, *BMI* body mass index, *AC* abdominal circumference

Comparisons of labour events and outcomes between the two groups are summarised in Table 3. There was a significantly lower intrapartum CD rate (16.0% [275/1722] vs. 26.7% [308/1154], *P* < 0.001), a higher proportion of amniotomy (53.4% [919/1722] vs. 42.3% [488/1154], *P* < 0.001) and oxytocin augmentation (79.5% [1369/1722] vs. 67.0% [773/1154], *P* < 0.001), and a higher incidence of intrapartum fever (≥38 °C) (23.3% [401/1722] vs. 8.5% [98/1154], *P* < 0.001) among women who received EA than among those who did not. However, the duration of labour (first and second stages), rates of assisted vaginal delivery and PROM, neonatal Apgar scores, birthweight, blood loss during the procedure, and length of hospital stay did not significantly differ between the two groups (Table 3).

**Table 3:** Labour events and outcomes of women in the two groups

Variables	Use of EA (*n* = 1722)	No use of EA (*n* = 1154)	*P* value
Intrapartum CD	275 (16.0)	308 (26.7)	< 0.001*
Assisted vaginal delivery	167 (9.7)	99 (8.6)	0.310
Length of first stage of labour (min)	629 ± 208	582 ± 246	0.076
Length of second stage of labour (min)	40.2 ± 27.1	37.4 ± 23.6	0.216
PROM	573 (33.3)	405 (35.1)	0.313
Augmentation with amniotomy	919 (53.4)	488 (42.3)	< 0.001*
Augmentation with oxytocin	1369 (79.5)	773 (67.0)	< 0.001*
Intrapartum fever (> 38 °C)	401 (23.3)	98 (8.5)	< 0.001*
VAS pain score (mm)			
Baseline	80.5 [77, 90]	78.7 [70, 83]	0.342
1 h after EA	20.9 [10, 30]	–	–
2 h after EA	33.5 [20, 50]	–	–
3 h after EA	39.8 [30, 50]	–	–
Satisfaction score of EA (mm)	90 [87.5, 100]	–	–
Blood loss (ml)	300 [300, 375]	300 [300, 300]	0.395
Hospital stays (d)	5 [4, 6]	5 [4, 6]	0.238
Birthweight (g)			
< 2500	5 (0.3)	7 (0.6)	0.462
2500–4000	1605 (93.2)	1064 (92.2)	
≥ 4000	112 (6.5)	82 (7.1)	
Apgar < 7 at 1 min	29 (1.7)	12 (1.0)	0.153
Apgar < 7 at 5 min	6 (0.4)	3 (0.3)	0.677
Cervical dilation at the time of intrapartum CD (cm)	(*n* = 275)	(*n* = 308)	
≤ 2	23 (8.4)	275 (89.3)	< 0.001*
3–4	175 (63.6)	30 (9.7)	
≥ 5	77 (28.0)	3 (1.0)	

Data were presented as mean ± standard deviation, n (%), or median [interquartile range]

Abbreviations: *EA* epidural analgesia, *CD* caesarean delivery, *PROM* premature rupture of membranes, *VAS* visual analogue scale

*P value< 0.05

The proportions of indications for CD in nulliparous women who did and did not receive EA are shown in Table 4. There was a lower probability of maternal request for CD among the women who received EA than among those who did not (3.9% [67/1722] vs. 10.5% [121/1154], *P* < 0.001). Regarding the reasons for the maternal request, there were significantly fewer complaints of pain (0.5% [8/1722] vs. 4.6% [53/1154], *P* < 0.001) and expectations of terminating delivery as soon as possible (1.3% [23/1722] vs. 3.6% [41/1154], *P* < 0.001) among the women who received EA than among those who did not. However, there was no difference between the two groups regarding concerns of foetal well-being as the reason for the maternal request (Table 4).

**Table 4:** Indications for intrapartum caesarean delivery in the two groups

Indication for intrapartum CD	Use of EA (*n* = 1722)	No use of EA (*n* = 1154)	*P* value
1. Fetal reasons (no oxytocin)	10 (0.6)	14 (1.2)	0.068
2. Dyst/IUA/ITT/FI	83 (4.8)	73 (6.3)	0.081
3. Dyst/IUA/ITT/OC	58 (3.4)	45 (3.9)	0.452
4. Dyst/IUA/PR	53 (3.1)	51 (4.4)	0.059
5. Dys/IUA/no oxytocin	1 (0.05)	1 (0.08)	0.776
6. Dys/EUA/CPD/Mal	3 (0.2)	3 (0.3)	0.621
7. Maternal request	67 (3.9)	121 (10.5)	< 0.001*
Unable to tolerate pain	8 (0.5)	53 (4.6)	< 0.001*
Expect to terminate delivery soon	23 (1.3)	41 (3.6)	< 0.001*
Worry about foetal condition	36 (2.1)	27 (2.3)	0.655

Data were presented as n (%)

*Abbreviations*: *CD* caesarean delivery, *Dyst* dystocia, *IUA* inefficient uterine action, *ITT* inability to treat adequately with oxytocin, *FI* fetal intolerance, *OC* over contracting, *PR* poor response, *EUA* efficient uterine action, *CPD* cephalopelvic disproportion, *Mal* malposition

*P value < 0.05

Due to the similar rates of indications for CD in the two groups, except for maternal request, we determined the association between EA and indications for intrapartum CD using the multivariate logistic regression analysis. EA administration was independently associated with a decreased risk of maternal request for CD (adjusted odds ratio [aOR], 0.30; 95% CI, 0.2–0.42; *P* < 0.001), while the use of oxytocin increased the risk of maternal request for CD (aOR, 2.34; 95%CI, 1.47–3.75; *P* < 0.001) (Table 5).

**Table 5:** Factors associated with maternal request in Group 1

Factors	Univariate logistic regression		Multivariate logistic regression	
	OR (95% CI)	*P*-value	aOR (95% CI)	*P*-value
Maternal age (y)	1.03 (0.97–1.09)	0.339	1.03 (0.97–1.10)	0.352
Gestational age (w)	0.95 (0.79–1.13)	0.538	0.93 (0.76–1.13)	0.449
BMI (kg/m^2^)	1.12 (1.06–1.24)	0.134	1.08 (1.04–1.21)	0.267
AC (cm)	1.04 (1.01–1.08)	0.225	0.99 (0.94–1.04)	0.691
Use of EA	0.35 (0.25–0.47)	< 0.001*	0.30 (0.22–0.42)	< 0.001*
Intrapartum fever	1.09 (0.75–1.60)	0.637	1.17 (0.76–1.79)	0.471
Oxytocin	2.13 (1.40–3.23)	< 0.001*	2.34 (1.47–3.75)	< 0.001*
Amniotomy	1.45 (1.07–1.95)	0.016*	1.13 (0.79–1.62)	0.504

*Abbreviations*: *BMI* body mass index, *AC* abdominal circumference, *EA* epidural analgesia, *OR* Odds ratio, *aOR* adjusted odds ratio, *CI* confidence interval

Intrapartum fever: body temperature ≥ 38.0 °C in labour

* *P* value < 0.05

## Discussion

This retrospective study showed a significant association between EA and a lower intrapartum CD rate in nulliparous women with singleton cephalic spontaneous term labour (Group 1). Despite a higher rate of intrapartum fever and a greater proportion of labour interventions (including oxytocin augmentation and amniotomy) among women who received EA than among those who did not, EA administration appeared to reduce the likelihood an intrapartum CD request by the mother. In contrast, no other indications for CD (such as foetal or obstetrical factors) were affected by EA administration.

The intrapartum CD rate in this study differed from that of another study conducted in Slovenia that applied the TGCS to examine the associations between EA and CD rates in different groups [15]. In that study, the women in Group 1 who received EA had a higher CD rate. Notably, the EA rate in Group 1 in the Slovenian study was extremely low at only 13.6% (9384/68790), compared to the 59.9% rate (1722/2876) in our study. In stark contrast, the overall use of intrapartum neuraxial analgesia (including epidural and combined spinal-epidural analgesia) in the United States in 2015 was 73.1% [16]. Additionally, incomplete statistics indicate that since 2019, > 80% of women in labour at our hospital have received EA. In contrast, the acceptance of EA in Slovenia is very low, possibly due to different cultural and local beliefs. Thus, we speculated that women requesting for EA after local anaesthesia might be experiencing excruciating dysfunctional (prolonged or obstructed) labour, rendering them more likely to accept a CD due to obstetrical factors, which may explain the differences in the study results. We plan to conduct a prospective study in the future to further verify the exact effects of EA on the intrapartum CD rate in different groups of the TGCS.

In previous studies, the influence of EA on the indications for intrapartum CD has not been appropriately evaluated; moreover, the indications for intrapartum CD have not been well defined and used consistently. Adopting standardised classification principles undoubtedly provides quality assurance in analysing obstetric events and outcomes [14]. The indications for CD performed in spontaneous labour are usually classified into foetal reasons and dystocia. However, as a sizable percentage of intrapartum CDs at our hospital were performed without medical indication, we added maternal request to the indication classifications (illustrated in Fig. 2). Based on a population-based maternal and child health surveillance investigation [17], the increase in CD rate in southeast China since 1998 has been primarily due to CD on maternal request (CDMR). Another survey study from Shanghai reported that 46% of women delivered by CDMR [18]. Notably, the situation in China is not a rare occurrence. A recent systematic review and meta-regression on global incidence of CDMR has reported that the absolute CDMR proportion ranges from 0.2–42% across the countries [19]. Among the five geographical regions involved in the review, the CDMR estimates in the Middle East were the highest (30.36%), followed by East Asia (17.51%). Furthermore, the CDMR rate in upper-middle-income regions is reportedly 11-times than that in high-income regions. The lack of a strict management system and the obstetricians’ acquiescence regarding CDMR in upper-middle-income country settings may be the potential reasons for it [20]. In China, fear of litigation, economic return, and the convenience of CD may be responsible for the obstetricians’ attitude towards CDMR [21]. A systematic review of 47 quantitative and 19 qualitative studies revealed that the women’s preferences for CD in China were mainly attributable to the fear of pain, distrustful relationships between providers and patients, and a deep-rooted belief that CD is a safer option [22].

Our study results demonstrated that the proportion of CDMR was significantly lower among the women who received EA than among those who did not. Further multivariate logistic regression analysis revealed that EA was negatively associated with CDMR. Based on the analysis of the reason for the maternal request for CD, we speculated that this negative association reflects effective labour pain control by EA. We observed that unbearable labour pain or a lack of progress in labour were more likely to result in women requesting to switch to CD during labour. Recently, Carvalho et al. [23] used a standardised questionnaire to evaluate the women’s pain preference. According to their data analysis, women preferred lower pain intensity at the expense of longer pain duration. Similarly, another study reported that availability of adequate pain relief during labour could decrease the maternal decision for CD by > 50%, especially in women expecting moderate and severe pain during their upcoming labour [24]. In this study, we regularly evaluated the pain intensity and actively treated breakthrough pain; accordingly, only 0.5% of women with EA requested a CD due to pain. Additionally, it is interesting to note that intrapartum conversion to CD occurred most frequently during the latent phase of the first stage of labour among the women who did not receive EA. In contrast, women who received EA usually switched to CD during the active phase. Based on the concerns regarding the safety of EA administration, and limitations according to the management mode of the delivery room, EA was generally administered when the cervix was dilated to 2 cm at our hospital. We propose that providing non-pharmacological pain relief interventions as an option during the early latent phase might contribute to a reduction in CDMR. However, further research is needed to confirm our conjecture.

In addition, oxytocin augmentation was revealed to increase the risk of a maternal request for CD on multivariate logistic regression analysis in this study. Although oxytocin administration can promote efficient uterine contractions and accelerate the labour course, it can also increase the pain intensity. Women perceiving enhanced labour pain could be more inclined to choose CD.

Regarding other indications for intrapartum CD, our study findings are consistent with those of a previous review in the Cochrane Database of Systematic Reviews [1], which showed no significant differences between women who did and did not receive EA in terms of CDs performed for foetal distress (relative ratio, 1.32; 95% CI, 0.97–1.79; 5753 women; 12 studies) and dystocia (relative ratio, 0.93; 95% CI, 0.79–1.11; 5938 women; 13 studies).

As for consequences of EA, more intrapartum interventions, including oxytocin augmentation and amniotomy, were used in women who received EA than in those who did not in this study. A prospective multicentre study also arrived at similar conclusions [25], as their EA group showed an increased risk of oxytocin augmentation (*P* = 0.030). Moreover, our study showed that more women experienced intrapartum fever (≥38 °C) after receiving EA, compared to those who did not, which is consistent with the results of a previous review [1]. Epidural-related maternal fever occurs in about 20% of labouring women with EA and does not occur in non-pregnant women or even pregnant women undergoing elective CD [26]. While the exact pathophysiological mechanism is not well understood, existing evidence suggests that epidural-related maternal fever may reflect a non-infectious inflammatory process [27]. A recent lab-based study conducted by Wohlrab et al. [28] showed that ropivacaine may induce the release of IL-6 and IL-8 cytokines and activate multiple proapoptotic and inflammatory signalling pathways (caspase, NFKB, and P38), triggering epidural-related maternal fever. Despite this, EA appeared to have no impact on assisted vaginal delivery rates and the duration of the first and second stages of labour in this study. This might be related to the lower concentrations of local anaesthetics involved in our study, and the treatment of breakthrough pain as required. As explained in previous systematic review [1], the association among EA, assisted vaginal delivery, and duration of labour have not been evaluated since 2005 due to modern EA approaches.

This study has several limitations. First, the women’s attitudes towards EA were not surveyed in this retrospective study. Although our institution provided pre-delivery counselling about pain-free birth and women were fully informed of the relative risks of CD, patient concerns regarding the safety and effectiveness of EA still exist. Second, we focused on the women in Group 1, and did not analyse other TGCS groups. The association between EA and indications for CD in different obstetric populations deserve further exploration. Third, the application of the TGCS on the impact of EA remains limited, and the conclusions drawn from this retrospective study remain incomplete and unilateral. It is essential to design a multicentre study to assess the impact of EA using a universal standard.

## Conclusions

Effective labour pain control with EA was associated with a lower risk of CD in nulliparous women with singleton cephalic spontaneous term labour (Group 1), which can be explained by a reduction in the likelihood of a maternal request for intrapartum CD. The use of EA had no significant association with foetal reasons and dystocia for CD. Further enhancing pre-labour health education, increasing the labour analgesia rate, and providing other options for pain relief may help to prevent primary CD in nulliparous women.

## Data Availability

All data generated and/or analysed during this study are not publicly available due to hospital’s policy, but are available from the corresponding author on reasonable request.

## Abbreviations

EA: Epidural anaesthesia
CD: Caesarean delivery
TGCS: 10-Group Classification System
RCD: Repeat caesarean delivery
VAS: Visual analogue scale
IUA: Inefficient uterine action
EUA: Efficient uterine action
ITT: Inability to treat adequately with oxytocin
FI: Foetal intolerance
OC: Uterus over-contracting
PR: Poor response to oxytocin
CPD: Cephalopelvic disproportion
BMI: Body mass index
AC: Abdominal circumference
PROM: Premature rupture of membranes
aOR: Adjusted odds ratio
CI: Confidence interval
CDMR: Caesarean delivery on maternal request
